# Cartilage Intermediate Layer Protein 2 Aggravates Hepatic Lipid Accumulation and Inflammation Through the IRE1α/XBP1 Pathway

**DOI:** 10.3390/ijms27031213

**Published:** 2026-01-25

**Authors:** Siqi Chen, Lun Dong, Yingying Shan, Zhili Chen, Yitao Xia, Jiaxin Liu, Dongfang Liu, Gangyi Yang, Mengliu Yang, Ke Li

**Affiliations:** Department of Endocrinology, The Second Affiliated Hospital, Chongqing Medical University, Chongqing 400010, China; 2023110671@stu.cqmu.edu.cn (S.C.); 2025140286@stu.cqmu.edu.cn (L.D.); 2024110711@stu.cqmu.edu.cn (Y.S.); 2023120783@stu.cqmu.edu.cn (Z.C.); 2024150889@stu.cqmu.edu.cn (Y.X.); 2025110826@stu.cqmu.edu.cn (J.L.); 300306@hospital.cqmu.edu.cn (D.L.); gangyiyang@hospital.cqmu.edu.cn (G.Y.); mengliu.yang@cqmu.edu.cn (M.Y.)

**Keywords:** CILP2, MASLD, endoplasmic reticulum stress, lipid metabolism, inflammation

## Abstract

Metabolic dysfunction-associated steatotic liver disease (MASLD) is the most common liver disease and is characterized by excessive lipid accumulation in hepatocytes. Endoplasmic reticulum (ER) stress and inflammation play important roles in hepatic lipid accumulation. Although CILP2 has been implicated in lipid metabolism, its role in MASLD remains unclear. Hepatic steatosis was induced in mice by a high-fat diet in this study. CILP2 was overexpressed in mouse livers and in vitro hepatocytes using the Ad-CILP2 adenovirus. CILP2 KO mice were also used in the experiments. Liver tissues and hepatocytes were collected for further analysis. CILP2 expression was upregulated in steatotic liver tissue and hepatocytes. CILP2 overexpression upregulated genes related to fatty acid synthesis (*Srebp-1c, Fasn, Acc, Scd1, and Cd36*), promoted lipid accumulation, and elevated the expression of proinflammatory cytokines (*Il6, Tnf, and Il1b)*. Conversely, CILP2 knockout reduced high-fat diet-induced hepatic steatosis and improved glucose metabolism. Mechanistically, CILP2 activated the IRE1α/XBP1 branch of the ER stress pathway, thereby promoting lipid synthesis and inflammation, effects that were partially alleviated by 4-PBA and STF-083010 treatments. Our findings indicate that CILP2 contributes to hepatic lipid accumulation and inflammation via the IRE1α/XBP1 pathway and may represent a potential therapeutic target for MASLD intervention.

## 1. Introduction

Metabolic dysfunction-associated steatotic liver disease (MASLD) includes a series of liver pathologies, starting with excessive accumulation of triglycerides (TGs) in hepatocytes, gradually progressing to metabolic dysfunction-associated steatohepatitis (MASH), and may even develop into cirrhosis and liver cancer [1,2]. Understanding its pathogenesis and identifying therapeutic targets remains of great significance.

The endoplasmic reticulum (ER) is the main organelle responsible for the correct folding of proteins. ER stress has been implicated in various metabolic diseases, including diabetes, cardiovascular disease, and MASLD [3]. Chronic ER stress in MASLD can aggravate hepatic steatosis. The three classic pathways of ER stress—protein kinase RNA-like endoplasmic reticulum kinase (PERK)–eukaryotic translation initiation factor 2α (eIF2α)–activating transcription factor 4 (ATF4), inositol-requiring enzyme 1α (IRE1α)–X-box binding protein 1 (XBP1), and ATF6 pathways—are all involved in lipid deposition in the liver [4]. Among them, IRE1α is the most conserved ER stress sensor. When ER stress occurs, IRE1α undergoes dimerization and autophosphorylation, activating its RNase activity and promoting the splicing of XBP1 mRNA to generate the transcription factor XBP1s [5]. XBP1s regulates genes involved in ER protein folding, secretion, ER-related degradation, and lipid synthesis. Hepatocyte-specific knockout of IRE1α or XBP1 has been shown to alleviate HFD-induced hepatic steatosis in mice [6,7]. In addition, IRE1α can promote inflammatory responses by recruiting TRAF2 and activating JNK and NF-κB signaling, leading to increased expression of cytokines such as TNF-α and IL-6, thereby aggravating hepatic injury [8].

Cartilage intermediate layer protein 2 (CILP2) is a secreted glycoprotein primarily expressed in the heart, skeletal muscle, and liver [9]. Several studies have reported associations between CILP2 gene polymorphisms and circulating lipid parameters, including total cholesterol, LDL-C, HDL-C, and TG levels [10,11,12,13]. A missense single nucleotide polymorphism (rs58542926) in the TM6SF2 gene located in the NCAN-CILP2 region has been linked to hepatic lipid accumulation and susceptibility to NAFLD (non-alcoholic fatty liver disease) in the Japanese population [14]. Our previous findings also showed that serum CILP2 concentrations were significantly elevated in overweight and obese individuals and were positively correlated with insulin resistance [9,15]. However, it is still unclear whether CILP2 plays a role in regulating liver lipid homeostasis. This study investigated the role of CILP2 in MASLD and examined the mechanism by which it regulates hepatic lipid accumulation.

## 2. Result

### 2.1. CILP2 Upregulated in Steatotic Liver and Hepatocytes

**Figure 1:**
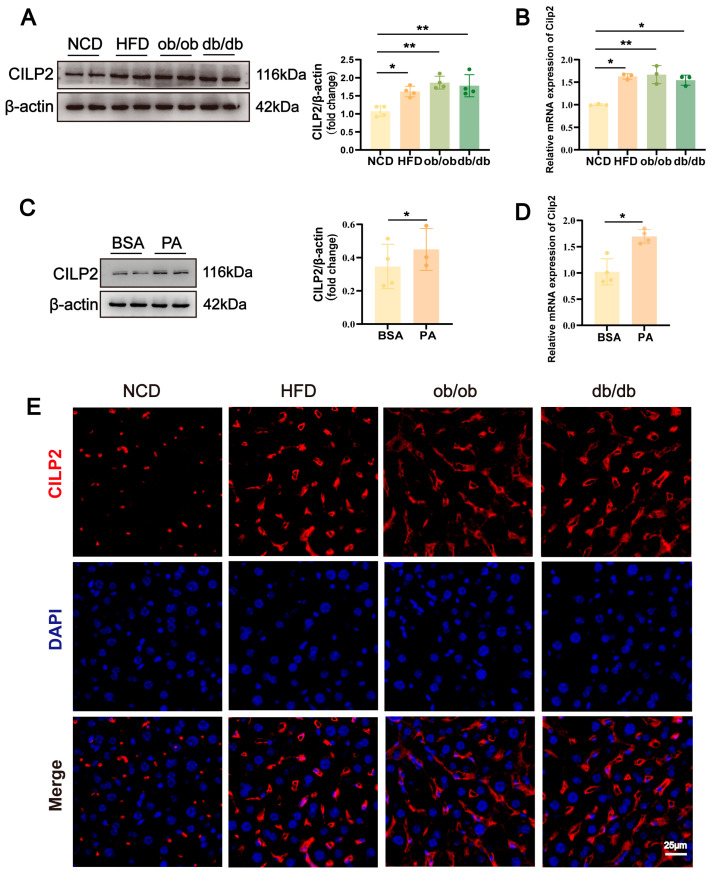
Hepatic CILP2 expression in mice models and hepatocytes. (**A**) Immunoblots (**left**) and quantification (**right**) of hepatic CILP2 protein level in mice models (*n* = 4). * *p* < 0.05, ** *p* < 0.01; one-way ANOVA with multiple comparisons. (**B**) qPCR analysis of hepatic Cilp2 mRNA expression in mice models (*n* = 3). * *p* < 0.05, ** *p* < 0.01; one-way ANOVA with multiple comparisons. (**C**) Immunoblots (**left**) and quantification (**right**) of CILP2 protein level in AML12 cells at 24 h post-intervention (*n* = 4). * *p* < 0.05; two-tailed unpaired Student’s *t* test. (**D**) qPCR analysis of Cilp2 mRNA expression in AML12 cells at 24 h post-intervention (*n* = 3). * *p* < 0.05; two-tailed unpaired Student’s *t* test. (**E**) Representative immunofluorescence images of CILP2 (red) and DAPI (blue) in liver from mice models. Scale bar: 25 μm.

The elevated hepatic CILP2 expression was observed in HFD-fed WT mice, ob/ob mice, and db/db mice (Figure 1A,B). Consistently, in AML12 cells, PA intervention induced higher CILP2 expression (Figure 1C,D). CILP2 was predominantly localized in the extracellular matrix (Figure 1E).

### 2.2. CILP2 Overexpression Enhanced Lipid Accumulation in Hepatocytes

**Figure 2:**
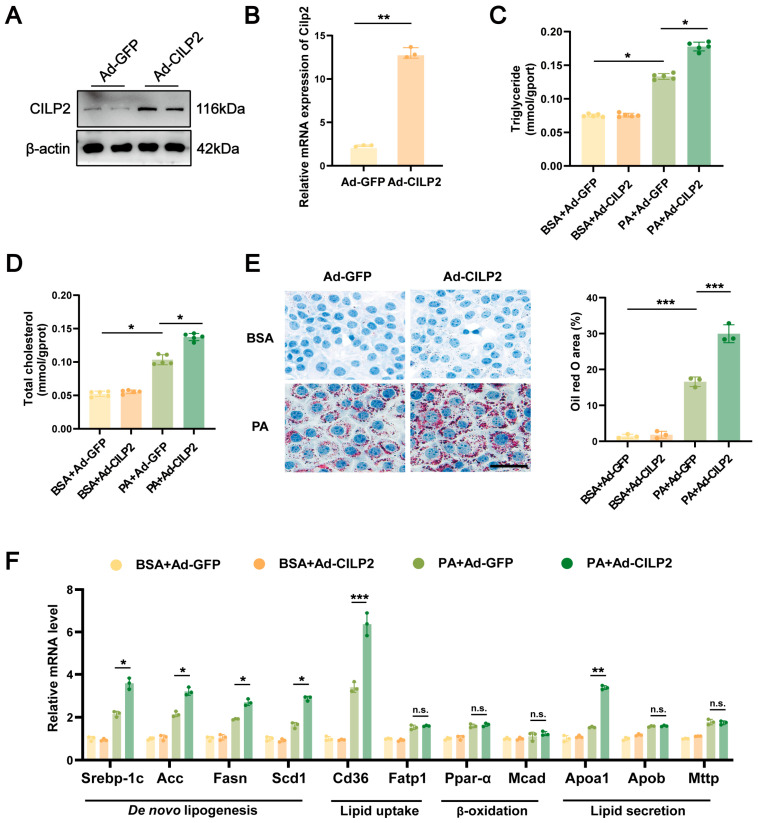
CILP2 overexpression enhanced lipid accumulation in PA-induced hepatocytes. (**A**) Immunoblots of CILP2 in the Ad-CILP2- or Ad-GFP-transfected AML12 cells (*n* = 4). (**B**) qPCR analysis of Cilp2 mRNA expression in the Ad-CILP2- or Ad-GFP-transfected AML12 cells (*n* = 3). ** *p* < 0.01; two-tailed unpaired Student’s *t* test. (**C**) Intracellular triglyceride content of the Ad-CILP2- or Ad-GFP-transfected AML12 cells post-BSA or PA intervention (*n* = 5). * *p* < 0.05; one-way ANOVA with multiple comparisons. (**D**) Intracellular cholesterol content of the Ad-CILP2- or Ad-GFP-transfected AML12 cells post-BSA or PA intervention (*n* = 5). * *p* < 0.05; one-way ANOVA with multiple comparisons. (**E**) Representative images (**left**) and quantification (**right**) of oil red O-stained AML12 cells post-BSA or PA intervention (*n* = 3). *** *p* < 0.001; one-way ANOVA with multiple comparisons. Scale bar = 50 μm. (**F**) qPCR analysis of genes expression of de novo lipogenesis, lipid uptake, β-oxidation, and lipid secretion in the AML12 cells post-BSA or PA intervention (*n* = 3). n.s., not significant; * *p* < 0.05, ** *p* < 0.01,*** *p* < 0.001; two-tailed unpaired Student’s *t* test.

CILP2-overexpressed hepatocytes (Figure 2A,B) exhibited significantly increased intracellular TG and TC levels following PA intervention (Figure 2C,D). Oil red O staining revealed increased lipid accumulation in the cells (Figure 2E). CILP2 overexpression also increased expressions of the genes involved in fatty acid synthesis and uptake (e.g., sterol regulatory element-binding protein 1c [*Srebp-1c*], acetyl-CoA carboxylase [*Acc*], fatty acid synthase [*Fasn*], stearoyl-CoA desaturase 1 [*Scd1*], and fatty acid translocase 36 [*Cd36*]), but had no effect on expressions of the genes related to fatty acid β-oxidation and lipid secretion (Figure 2F).

### 2.3. CILP2 KO Reduced Body Weight and Improved IR and Hepatic Steatosis in HFD-Fed Mice

**Figure 3:**
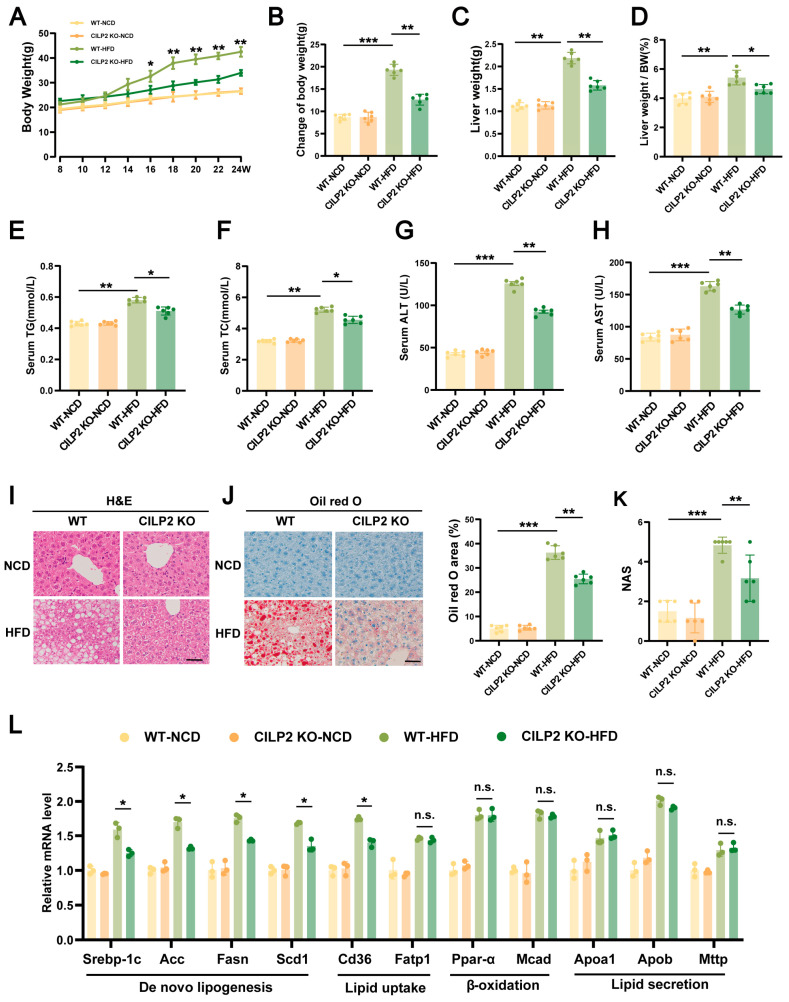
CILP2 knockout reduced body weight and improved IR and hepatic steatosis in HFD-fed mice. (**A**) Body weight of the WT or CILP2 KO mice fed an NCD or HFD for 16 weeks (*n* = 6) * *p* < 0.05, ** *p* < 0.01; one-way ANOVA with multiple comparisons. (**B**) Change in body weight of the indicated mice groups (*n* = 6). ** *p* < 0.01,*** *p* < 0.001; one-way ANOVA with multiple comparisons. (**C**,**D**) Liver weight (**C**) and liver weight/body weight ratio (**D**) of the indicated mice groups (*n* = 6). * *p* < 0.05, ** *p* < 0.01; one-way ANOVA with multiple comparisons. (**E**–**H**) Serum TG (**E**), TC (**F**), ALT (**G**), and AST (**H**) levels in the indicated mice groups (*n* = 6). * *p* < 0.05, ** *p* < 0.01,*** *p* < 0.001; one-way ANOVA with multiple comparisons. (**I**) Representative H&E staining of liver from the indicated mice groups (*n* = 6). Scale bar = 50 μm. (**J**) Representative images (**left**) and quantification (**right**) of oil red O-stained liver sections from the indicated mice groups (*n* = 6). ** *p* < 0.01,*** *p* < 0.001; one-way ANOVA with multiple comparisons. Scale bar = 50 μm. (**K**) NAS scores of the indicated mice groups (*n* = 6). ** *p* < 0.01,*** *p* < 0.001; one-way ANOVA with multiple comparisons. (**L**) qPCR analysis of genes expression of de novo lipogenesis, lipid uptake, β-oxidation, and lipid secretion in the indicated mice groups (*n* = 3). n.s., not significant; * *p* < 0.05; two-tailed unpaired Student’s *t* test.

CILP2 KO showed no significant effect on the metabolic parameters of the mice fed an NCD (Figure 3A–L). The HFD-fed CILP2 KO mice displayed lower body weight, liver weight, and liver-to-body weight ratio than the HFD-fed WT mice (Figure 3A–D). Serum TG and TC levels were also reduced (Figure 3E,F). In addition, GTT and ITT revealed a smaller area under the curve (AUC) in the HFD-fed CILP2 KO mice (Figure S1F,G), suggesting enhanced glucose metabolism. In the HFD-fed CILP2 KO mice, the livers were smaller and less pale (Figure S1E), with lower serum ALT and AST levels (Figure 3G,H). Reduced hepatic lipid accumulation, smaller lipid vacuoles, and decreased lipid droplet area were also observed (Figure 3I,J). The value of NAS was also significantly lower (Figure 3K). Moreover, the mRNA expressions of genes related to hepatic fatty acid synthesis and uptake were decreased (Figure 3L). This indicates that CILP2 deficiency mitigated HFD-induced hepatic steatosis.

### 2.4. CILP2 Overexpression Increased Body Weight and Promoted IR and Hepatic Steatosis in HFD-Fed Mice

**Figure 4:**
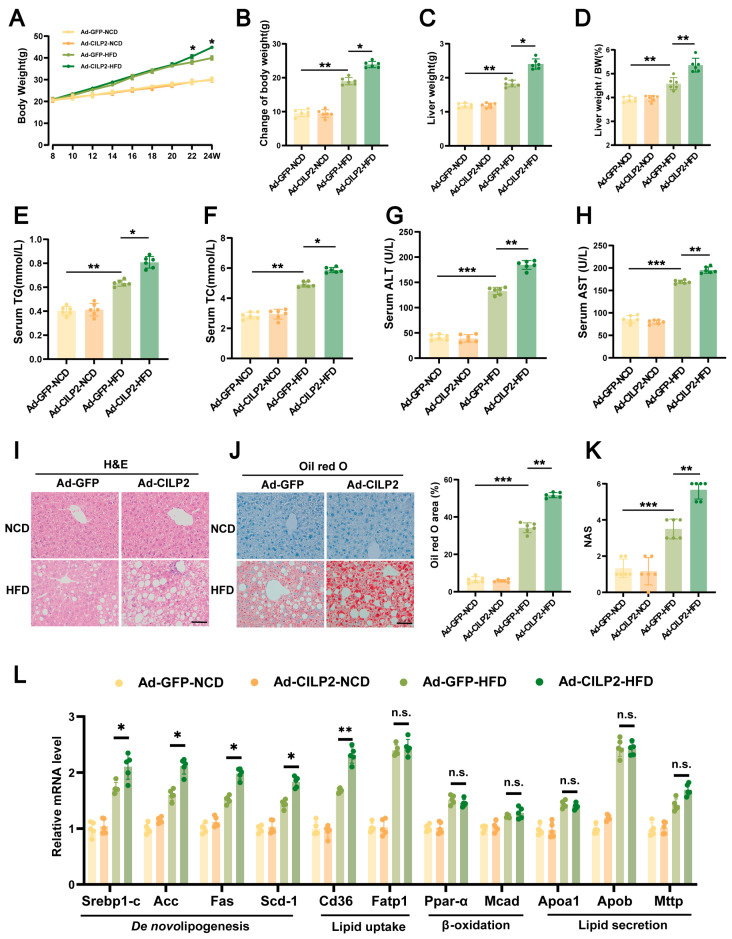
CILP2 overexpression increased body weight and promoted IR and hepatic steatosis in HFD-fed mice. (**A**,**B**) Body weight (**A**) and change in body weight (**B**) of the in the Ad-GFP or Ad-CILP2 mice fed an NCD or HFD for 16 weeks (*n* = 6). * *p* < 0.05, ** *p* < 0.01; one-way ANOVA with multiple comparisons. (**C**,**D**) Liver weight (**C**) and liver weight/body weight ratio (**D**) of the indicated mice groups (*n* = 6). * *p* < 0.05, ** *p* < 0.01; one-way ANOVA with multiple comparisons. (**E**–**H**) Serum TG (**E**), TC (**F**), ALT (**G**), and AST (**H**) levels in the indicated mice groups (*n* = 6). * *p* < 0.05, ** *p* < 0.01,*** *p* < 0.001; one-way ANOVA with multiple comparisons. (**I**) Representative H&E staining of liver from the indicated mice groups (*n* = 6). Scale bar = 50 μm. (**J**) Representative images (**left**) and quantification (**right**) of oil red O-stained liver sections from the indicated mice groups (*n* = 6). ** *p* < 0.01,*** *p* < 0.001; one-way ANOVA with multiple comparisons. Scale bar = 50 μm. (**K**) NAS scores of the indicated mice groups (*n* = 6). ** *p* < 0.01,*** *p* < 0.001; one-way ANOVA with multiple comparisons. (**L**) qPCR analysis of genes expression of de novo lipogenesis, lipid uptake, β-oxidation, and lipid secretion in the indicated mice groups (*n* = 5). n.s., not significant; * *p* < 0.05, ** *p* < 0.01; two-tailed unpaired Student’s *t* test.

To further evaluate the role of CILP2 in hepatic lipid metabolism, Ad-CILP2 was administered to WT mice via tail vein injection (Figure S2A–C). In the HFD-fed Ad-CILP2 mice, increased body weight, liver weight, and liver-to-body weight ratio were observed (Figure 4A–D), along with elevated serum TG and TC levels (Figure 4E,F), increased AUCs in GTT and ITT (Figure S2E,F), enlarged and pale livers (Figure S2D), and raised serum ALT and AST levels (Figure 4G,H). Elevated hepatic lipid accumulation, larger lipid vacuoles, and increased lipid droplet area (Figure 4I,J) were also observed. The NAS was also elevated (Figure 4K). The expressions of genes involved in hepatic fatty acid synthesis and uptake was upregulated (Figure 4L). These results indicate that CILP2 overexpression aggravated HFD-induced hepatic steatosis.

### 2.5. ER Stress in CILP2-Mediated Hepatic Lipid Accumulation

**Figure 5:**
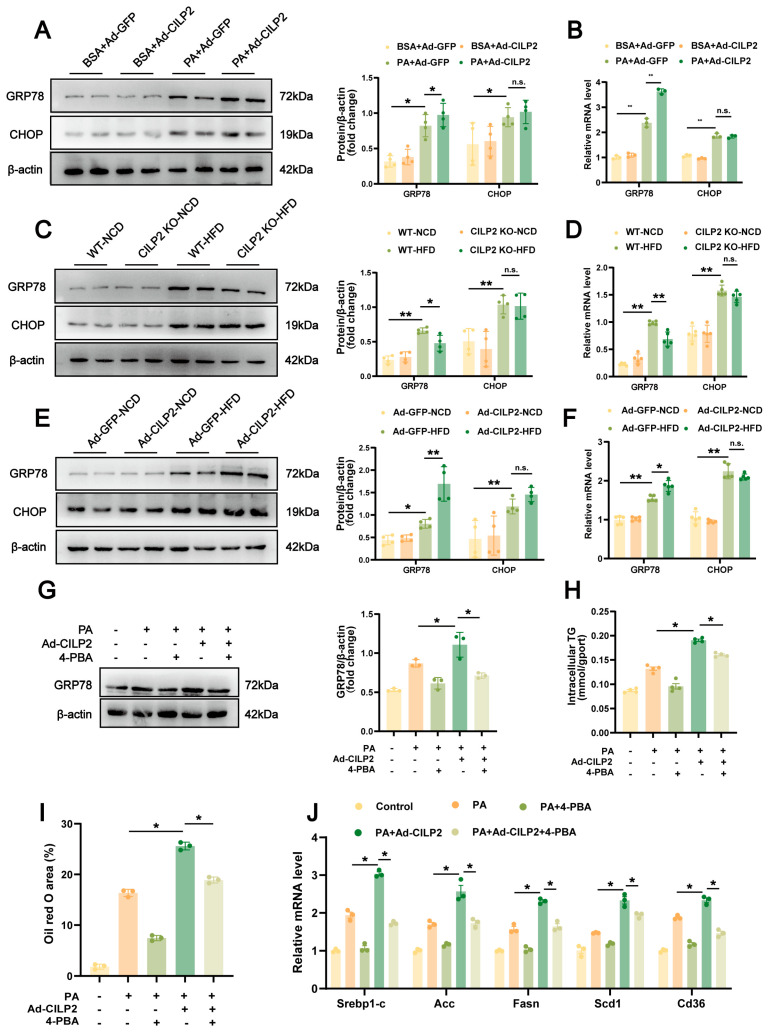
ER stress in CILP2-mediated hepatic lipid accumulation in vitro and vivo. (**A**) Immunoblots (**left**) and quantification (**right**) of GRP78 and CHOP protein levels in Ad-CILP2- or Ad-GFP-transfected AML12 cells (*n* = 4). n.s., not significant; * *p* < 0.05; one-way ANOVA with multiple comparisons. (**B**) qPCR analysis of GRP78 and CHOP mRNA expressions in Ad-CILP2- or Ad-GFP-transfected AML12 cells (*n* = 3). n.s., not significant; ** *p* < 0.01; one-way ANOVA with multiple comparisons. (**C**) Immunoblots (**left**) and quantification (**right**) of GRP78 and CHOP protein levels in the WT or CILP2 KO mice fed an NCD or HFD for 16 weeks (*n* = 4). n.s., not significant; * *p* < 0.05, ** *p* < 0.01; one-way ANOVA with multiple comparisons. (**D**) qPCR analysis of GRP78 and CHOP mRNA expressions in the WT or CILP2 KO mice fed an NCD or HFD for 16 weeks (*n* = 3). n.s., not significant; ** *p* < 0.01; one-way ANOVA with multiple comparisons. (**E**) Immunoblots (**left**) and quantification (**right**) of GRP78 and CHOP protein levels in Ad-GFP or Ad-CILP2 mice fed an NCD or HFD for 16 weeks (*n* = 4). n.s., not significant; * *p* < 0.05, ** *p* < 0.01; one-way ANOVA with multiple comparisons. (**F**) qPCR analysis of GRP78 and CHOP mRNA expressions in Ad-GFP or Ad-CILP2 mice fed an NCD or HFD for 16 weeks (*n* = 3). n.s., not significant; * *p* < 0.05, ** *p* < 0.01; one-way ANOVA with multiple comparisons. (**G**) Immunoblots (**left**) and quantification (**right**) of GRP78 protein level in the AML12 cells at 4 h after 0.5 mM 4-PBA intervention (*n* = 3). * *p* < 0.05; two-tailed unpaired Student’s *t* test. (**H**) Intracellular TG content in the AML12 cells at 4 h after 0.5 mM 4-PBA intervention (*n* = 4). * *p* < 0.05; two-tailed unpaired Student’s *t* test. (**I**) Quantification of oil red O-stained AML12 cells at 4 h after 0.5 mM 4-PBA intervention (*n* = 3). * *p* < 0.05; two-tailed unpaired Student’s *t* test. (**J**) qPCR analysis of genes expression of Srebp-1c, Fasn, Acc, Scd1, and Cd36 in AML12 cells at 4 h after 0.5 mM 4-PBA intervention (*n* = 3). * *p* < 0.05; two-tailed unpaired Student’s *t* test.

In AML12 cells, CILP2 overexpression further increased PA-induced GRP78 expression, while no significant change was observed in CHOP (Figure 5A,B). In vivo, hepatic GRP78 expression was also further increased in Ad-CILP2 mice and reduced in CILP2 KO mice. The CHOP expression was not significantly altered (Figure 5C–F). 4-PBA intervention reversed the enhancing effect of CILP2 overexpression on PA-induced lipid accumulation (Figure 5G–I), and reduced the expression of genes involved in fatty acid synthesis and uptake (Figure 5J). These findings suggest that ER stress, particularly through GRP78 activation, was involved in the regulation of CILP2 in hepatic lipid accumulation.

### 2.6. CILP2 Enhanced Hepatic Lipid Accumulation by Promoting the IRE1α/XBP1 Pathway

**Figure 6:**
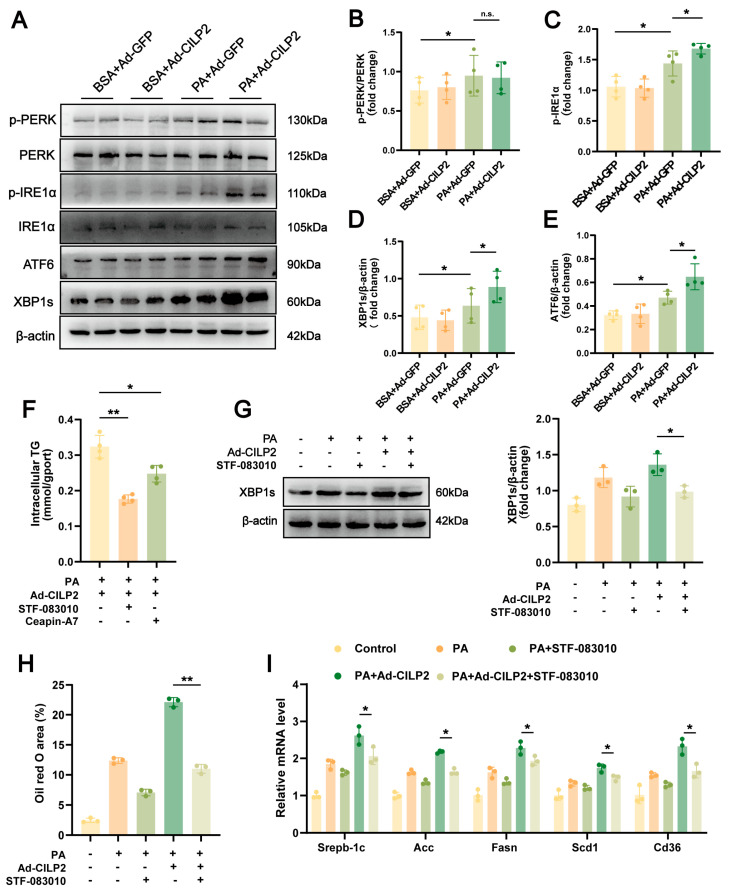
CILP2 overexpression enhanced hepatic lipid accumulation by promoting the IRE1α/XBP1 pathway. (**A**–**E**) Immunoblots (**A**) and quantification of p-PERK (**B**), p-IRE1α (**C**), XBP1s (**D**), and ATF6 (**E**) protein levels in Ad-CILP2- or Ad-GFP-transfected AML12 cells (*n* = 4). n.s., not significant; * *p* < 0.05; one-way ANOVA with multiple comparisons. (**F**) Intracellular TG content in the AML12 cells at 1 h after 50 μM STF-083010 or 6 μM Ceapin-A7 intervention (*n* = 4). * *p* < 0.05, ** *p* < 0.01; one-way ANOVA with multiple comparisons. (**G**) Immunoblots (**left**) and quantification (**right**) of XBP1s protein level in the AML12 cells at 1 h after 50 μM STF-083010 intervention (*n* = 3). * *p* < 0.05; two-tailed unpaired Student’s *t* test. (**H**) Quantification of oil red O-stained AML12 cells at 1 h after 50 μM STF-083010 intervention (*n* = 3). ** *p* < 0.01; two-tailed unpaired Student’s *t* test. (**I**) qPCR analysis of genes expression of *Srebp-1c, Fasn, Acc, Scd1, and Cd36* in AML12 cells at 1 h after 50 μM STF-083010 intervention (*n* = 3). * *p* < 0.05; two-tailed unpaired Student’s *t* test.

To further clarify the ER stress pathway involved in CILP2-mediated hepatic lipid accumulation, both in vitro and in vivo experiments were performed. In AML12 cells, CILP2 overexpression increased the protein levels of ATF6, phosphorylated IRE1α (p-IRE1α), and spliced XBP1 (XBP1s) induced by PA, while no significant change occurred in phosphorylated PERK (p-PERK) (Figure 6A–E). These results were confirmed in vivo (Figure S3). To verify the pathway, CILP2-overexpressed AML12 cells were treated with STF-083010, a selective inhibitor of the IRE1α pathway, or Ceapin-A7, a selective blocker of the ATF6 pathway. Both inhibitors reduced TG levels in PA-induced AML12 cells, with STF-083010 showing a more pronounced effect (Figure 6F). STF-083010 intervention alleviated the enhancing effect of CILP2 overexpression on PA-induced lipid accumulation (Figure 6G,H). Additionally, the expressions of genes involved in fatty acid synthesis and uptake was reduced (Figure 6I). These results suggest that CILP2 promoted hepatic lipid accumulation primarily through the IRE1α/XBP1 signaling pathway.

### 2.7. CILP2 Regulated Hepatic Inflammatory Responses Through ER Stress

**Figure 7:**
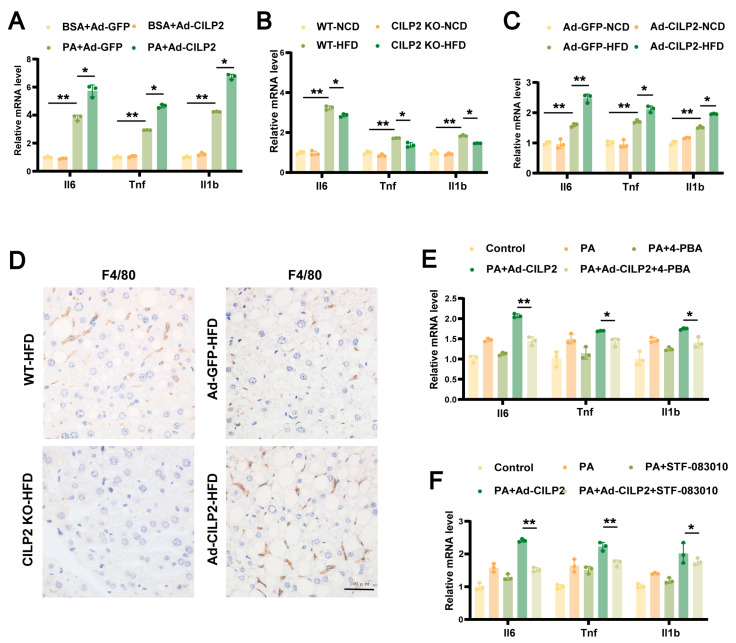
CILP2 regulated hepatic inflammatory response through ER stress. (**A**) qPCR analysis of *Il6, Tnf, and Il1b* mRNA expressions in Ad-CILP2- or Ad-GFP-transfected AML12 cells (*n* = 3). * *p* < 0.05, ** *p* < 0.01; one-way ANOVA with multiple comparisons. (**B**) qPCR analysis of *Il6, Tnf, and Il1b* mRNA expressions in the WT or CILP2 KO mice fed NCD or HFD for 16 weeks (*n* = 3). * *p* < 0.05, ** *p* < 0.01; one-way ANOVA with multiple comparisons. (**C**) qPCR analysis of *Il6, Tnf, and Il1b* mRNA expressions in Ad-GFP or Ad-CILP2 mice fed NCD or HFD for 16 weeks (*n* = 3). * *p* < 0.05, ** *p* < 0.01; one-way ANOVA with multiple comparisons. (**D**) Representative images of immunohistochemical staining for F4/80 in liver sections from the indicated mice groups. Scale bar = 100 μm. (**E**) qPCR analysis of *Il6, Tnf, and Il1b* mRNA expressions in AML12 cells at 4 h after 0.5 mM 4-PBA intervention (*n* = 3). * *p* < 0.05, ** *p* < 0.01; two-tailed unpaired Student’s *t* test. (**F**) qPCR analysis of *Il6, Tnf, and Il1b* mRNA expressions in AML12 cells at 1 h after 50 μM STF-083010 intervention (*n* = 3). * *p* < 0.05, ** *p* < 0.01; two-tailed unpaired Student’s *t* test.

CILP2 overexpression in hepatocytes increased mRNA expression of *Il6, Tnf, and Il1b* (Figure 7A). Consistently, CILP2 KO mice showed a reduction in hepatic macrophage infiltration, as well as decreased proinflammatory cytokine expressions compared to the WT mice (Figure 7B,D). In contrast, Ad-CILP2 mice exhibited higher macrophage density and elevated cytokine expressions (Figure 7C,D). Moreover, treatment with 4-PBA or STF-083010 attenuated the upregulation of proinflammatory genes induced by CILP2 (Figure 7E,F). These findings suggest that CILP2 promoted hepatic inflammation and lipid accumulation through the IRE1α/XBP1 pathway.

## 3. Discussion

MASLD, previously referred to as non-alcoholic fatty liver disease (NAFLD), is one of the most prevalent causes of chronic liver disease and represents a growing global health concern [2]. In this study, we demonstrated that CILP2 contributes to the progression of MASLD, and that its effects were at least partly mediated through activation of the IRE1α/XBP1 branch of the ER stress pathway.

Upregulated CILP2 levels in liver tissue have been observed in metabolic disorder models and cells. Wu et al. reported that circulating CILP2 levels increased in individuals with impaired glucose tolerance [9]. Similarly, Li et al. found that serum CILP2 concentrations were increased in overweight and obese individuals. Bioinformatic analyses indicated that CILP2-related genes are mainly associated with lipid metabolism and insulin resistance [15]. Hu et al. also reported that CILP2 promotes lipid uptake and foam cell formation [16]. CILP2-KO improved metabolic parameters, including weight gain, lipid profiles, hepatic lipid accumulation, and insulin resistance in HFD-fed mice. Conversely, CILP2 overexpression exacerbated these parameters. This supports a “promoting” role of CILP2 in liver metabolic disorders.

In vitro and vivo, CILP2 overexpression upregulated the expression of genes involved in hepatic fatty acid synthesis and uptake. This mechanism aligns with the known role of ER stress in hepatic lipid metabolism. Chronic unresolved ER stress and sustained activation of the unfolded protein response (UPR) have been shown to promote MASLD progression by enhancing lipogenesis, impairing mitochondrial function, and disrupting insulin signaling pathways [17,18,19]. The IRE1α/XBP1 pathway is an important hub for regulating liver lipid metabolism [20,21]. Wang et al. reported that IRE1α/XBP1s can promote VLDL assembly and secretion and maintain liver lipid homeostasis [22]. However, under abnormal metabolic conditions, this mechanism may be disrupted or reversed. Mao et al. reported that hepatic IRE1a is hyperactivated in the state of obesity [23]. Silencing XBP1s reduced fructose-induced transcriptional activity of SREBP-1c, ACC, and FASN in HepG2 cells, and lipid content was also downregulated [24]. In the present study, CILP2 upregulated GRP78, p-IRE1α, and XBP1s expression in HFD-fed mice. Although CILP2 did not significantly regulate the CHOP or PERK pathways in this study, it may still play an auxiliary role through other UPR branches under different conditions.

Inflammation is a key driver of tissue damage, fibrosis, and liver dysfunction in MASLD/MASH. ER stress contributes directly to upregulate proinflammatory factors through the IRE1α–TRAF2–JNK/NF-κB pathways, thereby promoting immune cell infiltration via releasing damage-associated molecular or apoptosis [4,25,26]. Previous studies showed that the IRE1α–XBP1 pathway can regulate NF-κB–mediated proinflammatory responses in macrophages, enhancing the expression of IL-1β and TNFα [27]. CILP2 overexpression induced expression of IL-6, TNFα, and IL-1β, along with enhanced hepatic macrophage infiltration. Conversely, CILP2 deficiency suppressed hepatic inflammatory cytokine expression and macrophage infiltration. Inhibition of both general ER stress and IRE1α-specific splicing partially reversed the hepatic lipid accumulation induced by CILP2 overexpression and attenuated the upregulation of hepatic proinflammatory factors, demonstrating that effects of CILP2 are mediated, at least in part, by the IRE1α/XBP1 pathway. However, the receptor or signaling axis linking CILP2 to IRE1α/XBP1 activation remains unknown and warrants further investigation.

## 4. Materials and Methods

### 4.1. Animal Models and Treatment

C57BL/6J mice (wild-type, WT) were purchased from the Laboratory Animal Center of Chongqing Medical University, and db/db and ob/ob mice were purchased from GemPharmatech Co., Ltd. (Nanjing, China). CILP2 knockout (CILP2 KO, C57BL/6J background) mice were purchased from Viewsolid Biotech Co., Ltd. (Beijing, China). The mice were housed in a 12 h light/12 h dark cycle at 23 ± 3 °C and 30–70% humidity, with free access to food and water. Eight-week-old male mice were used in the experiment and fed a high-fat diet (HFD, 20% protein; 60% fat; 20% carbohydrate, Research Diets, New Brunswick, NJ, USA) or normal chow diet (NCD, 20.6% protein; 12% fat; 67.4% carbohydrate) for 16 weeks. CILP2 adenovirus (Ad-CILP2) and control virus (Ad-GFP) were constructed by Hanbio Biotechnology Co., Ltd. (Shanghai, China). For hepatic overexpression of CILP2, Ad-CILP2 (1 × 10^9^ PFU) was injected into the tail vein of mice after 12 weeks of feeding, once a week for 4 weeks, and Ad-GFP was injected into control mice [28]. After fasting for 12 h, the mice were sacrificed by thoracotomy under CO_2_ anesthesia. Whole blood samples were collected by cardiac puncture and centrifuged at 3000× *g* and 4 °C for 20 min to obtain serum. The liver was immediately weighed and stored at −80 °C until further analysis.

### 4.2. Cell Culture and Treatment

The AML12 cell line was provided by Procell Life Science & Technology Co., Ltd. AML12 cells were cultured in high-glucose DMEM supplemented with 10% fetal bovine serum (FBS, F801-050, BDBIO, Shanghai, China) and 1% penicillin/streptomycin (G4016, Servicebio, Wuhan, China) in a CO_2_ incubator (5% CO_2_, 37 °C). Cells were treated with 0.4 mM palmitic acid (PA, P0500, Sigma, Burlington, MA, USA) for 24 h to induce lipid accumulation, which was visualized using oil red O staining (WLA055a, Wanleibio, Shenyang, China). Intracellular TG and total cholesterol (TC) contents were quantified using commercial assay kits (Nanjing Jiancheng Bioengineering Institute, Nanjing, China). The Ad-CILP2 and Ad-GFP adenoviruses were transfected into AML12 cells, and the cells were collected for subsequent measurement. To inhibit endoplasmic reticulum stress, cells were pretreated with 4-phenylbutyric acid (4-PBA; HY-A0281, MCE, China) at 0.5 mM (dissolved in DMSO) for 4 h before PA exposure [29]. Cells were also pretreated with the IRE1α inhibitor STF-083010 (50 μM; HY-15845, MCE, Shanghai, China) or the ATF6 pathway inhibitor Ceapin-A7 (6 μM; HY-108434, MCE, China) 1 h before PA intervention [30,31].

### 4.3. In Vivo Experiment

Food intake and body weight were monitored every other day throughout the experiment. The liver-to-body weight ratio (LW/BW) was calculated as LW/BW (%) = liver weight(g)/body weight at sacrifice (g) × 100. Glucose tolerance tests (GTTs) and insulin tolerance tests (ITTs) were conducted one week before the end of the study. For the GTTs, mice were fasted for 12 h and intraperitoneally injected with glucose (1.5 g/kg). For the ITTs, mice were fasted for 6 h and injected with insulin (1 U/kg). Blood glucose levels were measured at 0, 15, 30, 60, and 120 min after injection using a glucometer. Serum aspartate aminotransferase (AST), alanine aminotransferase (ALT), triglyceride (TG), and total cholesterol (TC) levels were determined using commercial assay kits (Nanjing Jiancheng Bioengineering Institute, China) according to the manufacturer’s instructions.

### 4.4. Histological Analysis

Mouse liver tissues were fixed, embedded, sectioned, and stained with hematoxylin and eosin (H&E) (C0105S, Beyotime, Shanghai, China) for hepatic morphology. Macrophage infiltration was assessed by immunohistochemical staining with F4/80 antibody. Lipid accumulation in liver tissue was examined by oil red O staining. The light microscope was used for visualization and imaging. The non-alcoholic fatty liver disease activity score (NAS) was determined for evaluating the degree of steatosis, lobular inflammation, and hepatocellular ballooning [32].

### 4.5. Immunohistochemistry and Immunofluorescence

Experiments were performed following the standard procedures. Fluorescence images were captured using a fluorescence microscope (Olympus BX53, Tokyo, Japan). For immunohistochemical staining, images were examined by a light microscope. All the images were analyzed by Image J software (Fiji distribution, version 1.53c; National Institutes of Health, USA).

### 4.6. Western Blot Analysis

Total proteins were extracted, separated by SDS-PAGE, and transferred onto membranes (IPVH00010, Merck, Darmstadt, Germany). The membranes were incubated overnight at 4 °C with specific primary antibodies and then with appropriate HRP-conjugated secondary antibodies. Protein bands were visualized using an enhanced chemiluminescence detection system. Details of all antibodies are provided in Supplementary Table S1.

### 4.7. RNA Extraction and Quantitative RT-PCR Analysis (qRT-PCR)

Total RNA was isolated from cells and tissues using TRIzol reagent (TaKaRa, Tokyo, Japan). Complementary DNA (cDNA) was synthesized from total RNA using a cDNA synthesis kit (RK26500, ABclonal, Woburn, MA, USA). qRT-PCR was performed using the ABScript II One Step SYBR Green RT-qPCR Kit (RK20404, ABclonal, USA). Primer sequences are listed in Supplementary Table S2.

### 4.8. Statistical Analysis

The data are displayed as mean ± SEM. A two-tailed Student’s *t* test was conducted to compared significance between two groups, and one-way ANOVA followed by Tukey’s post hoc test was performed to compare significance among three or more than three groups. A value of *p* < 0.05 was considered statistically significant. Graphpad prism 8.0 software was used to perform statistical analysis.

## 5. Conclusions

In summary, our study established that CILP2 overexpression exacerbated hepatic lipid accumulation and inflammatory responses by activating the IRE1α/XBP1 pathway. These findings position CILP2 as a potential therapeutic target for MASLD, and targeting this pathway may represent a promising therapeutic target.

## Data Availability

The original contributions presented in this study are included in the article/Supplementary Materials. Further inquiries can be directed to the corresponding author.

## Supplementary Materials

The following supporting information can be downloaded at https://www.mdpi.com/article/10.3390/ijms27031213/s1.

## Abbreviations

MASLD	Metabolic dysfunction-associated fatty liver disease
MASH	Metabolic dysfunction-associated steatohepatitis
ER	Endoplasmic reticulum
SREBP-1c	Sterol regulatory element-binding protein-1c
FASN	Fatty acid synthase
ACC	Acetyl-CoA carboxylase
SCD1	Stearoyl-CoA desaturase 1
CD36	Cluster of differentiation 36
IL-6	Interleukin-6
TNFα	Tumor necrosis factorα
IL-1β	Interleukin-1β
CILP2	Cartilage intermediate layer protein 2
TG	Triglyceride
TC	Total cholesterol
AST	Aspartate amino transferase
ALT	Alanine amino transferase
PERK	Protein kinase RNA-like endoplasmic reticulum kinase
eIF2α	Eukaryotic translation initiation factor 2α
ATF4	Activating transcription factor 4
IRE1α	Inositol-requiring enzyme 1α
XBP1	X-box binding protein 1
ATF6	Activating transcription factor 6
GRP78	Glucose-regulated protein 78
CHOP	C/EBP homologous protein
PA	Palmitic acid
BSA	Bovine serum albumin
GTT	Glucose tolerance test
ITT	Insulin tolerance test
NAS	Non-alcoholic fatty liver disease activity score
IR	Insulin resistance
HFD	High fat diet
NCD	Normal chow diet
AUC	Area under the curve
