# Supplementary material for: Cartilage Intermediate Layer Protein 2 Aggravates Hepatic Lipid Accumulation and Inflammation Through the IRE1α/XBP1 Pathway
Source: Int J Mol Sci. 2026 Jan 25;27(3):1213. doi: 10.3390/ijms27031213 (PMC12898282; doi:10.3390/ijms27031213)
Supplement: Supplementary file 1 [file ijms-27-01213-s001.zip › ijms-4061056-supplementary.pdf]

Supplementary Information

Cartilage intermediate layer protein 2 aggravates hepatic lipid accumulation and inflammation through the IRE1α/XBP1 pathway

Supplementary Table S1. Antibodies used for Western bolt.

Antibodies	Dilution ratio	Company	Lot
CILP2	WB 1:100	Santa Cruz	sc-390297
GRP78	WB 1:1000	Zenbio	200310-4F11
CHOP	WB 1:1000	Abiowell	AWA10322
p-PERK	WB 1:500	Abiowell	AWA40275
PERK	WB 1:1000	Upingbio	YP-mAb-14901
p-IRE1α	WB 1:500	Abiowell	AWA41548
IRE1α	WB 1:1000	Upingbio	YP-mAb-03498
ATF6	WB 1:1000	Upingbio	YP-mAb-12193
XBP1s	WB 1:1000	Proteintech	83959-5-RR
β-actin	WB 1:2000	Servicebio	GB11001-100

Supplementary Table S2. Sequences of Primers used for qRT-PCR.

Company	Primers	Sequences
Accurate Biotechnology(Hunan)	SREBP1-c (mouse)	Forward: 5'-TGACCCGGCTATTCCGTGA-3'
		Reverse: 5'-CTGGGCTGAGCAATACAGTTC-3'
Accurate Biotechnology(Hunan)	ACC (mouse)	Forward: 5'-ATGGCGGAATGGTCTCTTTC-3'
		Reverse: 5'-TGGGGACCTTGTCTTCATCAT-3'
Accurate Biotechnology(Hunan)	FAS (mouse)	Forward: 5'-GGAGGTGGTGATAGCCGGTAT-3'
		Reverse: 5'-TGGGTAATCCATAGAGCCCAG-3'
Accurate Biotechnology(Hunan)	SCD-1 (mouse)	Forward:5'-TTCTTGCGATACACTCTGGTGC-3'
		Reverse: 5'-CGGGATTGAATGTTCTTGTCGT-3'
Accurate Biotechnology(Hunan)	CD-36 (mouse)	Forward: 5'-ATGGGCTGTGATCGGAACTG-3'
		Reverse: 5'-TTTGCCACGTCATCTGGGTTT-3'
Accurate Biotechnology(Hunan)	FATP1 (mouse)	Forward: 5'-CGCTTTCTGCGTATCGTCTG-3'
		Reverse: 5'-GATGCACGGGATCGTGTCT-3'
Accurate Biotechnology(Hunan)	PPAR-α(mouse)	Forward: 5'-AGAGCCCCATCTGTCCTCTC-3'
		Reverse:5'-ACTGGTAGTCTGCAAAACCAAA-3'
Accurate	MCAD (mouse)	Forward: 5'-AGGGTTTAGTTTTGAGTTGACGG-3'

Biotechnology(Hunan)		Reverse: 5'-CCCCGCTTTTGTCATATTCCG-3'
Accurate Biotechnology(Hunan)	APOA1 (mouse)	Forward: 5'-GGCACGTATGGCAGCAAGAT-3'<br>Reverse:5'-CCAAGGAGGAGGATTCAAACTG-3'
Accurate Biotechnology(Hunan)	APOB (mouse)	Forward: 5'-AAGCACCTCCGAAAGTACGTG-3'<br>Reverse:5'-CTCCAGCTCTACCTTACAGTTGA-3'
Accurate Biotechnology(Hunan)	MTTP (mouse)	Forward: 5'-CTCTTGGCAGTGCTTTTTCTCT-3'<br>Reverse: 5'-GAGCTTGTATAGCCGCTCATT-3'
Accurate Biotechnology(Hunan)	CILP2 (mouse)	Forward: 5'-GAATTCATGAAGAGTAAAAAGCCCCTG-3'<br>Reverse:5'-GTCGACCTACAGCAGGACCTTGGTG-3'
Tsingke Biotechnology	GRP78 (mouse)	Forward: 5'-ACTTGGGGACCACCTATTCCT-3'<br>Reverse: 5'-ATCGCCAATCAGACGCTCC-3'
Tsingke Biotechnology	CHOP(mouse)	Forward: 5'-CTGGAAGCCTGGTATGAGGAT-3'<br>Reverse: 5'-CAGGGTCAAGAGTAGTGAAGGT-3'
Accurate Biotechnology(Hunan)	IL-6 (mouse)	Forward: 5'-CTGCAAGAGACTTCCATCCAG-3'<br>Reverse: 5'-AGTGGTATAGACAGGTCTGTTGG-3'
Accurate Biotechnology(Hunan)	TNF-α (mouse)	Forward: 5'-CCTCTTCTCATTCCTGCTTGTGG-3'<br>Reverse: 5'-GGCCATTTGGGAACTTCTCATC-3'
Accurate Biotechnology(Hunan)	IL-1β (mouse)	Forward: 5'-GAAATGCCACCTTTTGACAGTG-3'<br>Reverse: 5'-TGGATGCTCTCATCAGGACAG-3'
Accurate Biotechnology(Hunan)	β-actin (mouse)	Forward: 5'-GCTGTCCCTGTATGCCTCT-3'<br>Reverse: 5'-GATGTCACGCACGATTTCC-3'

Supplementary figures

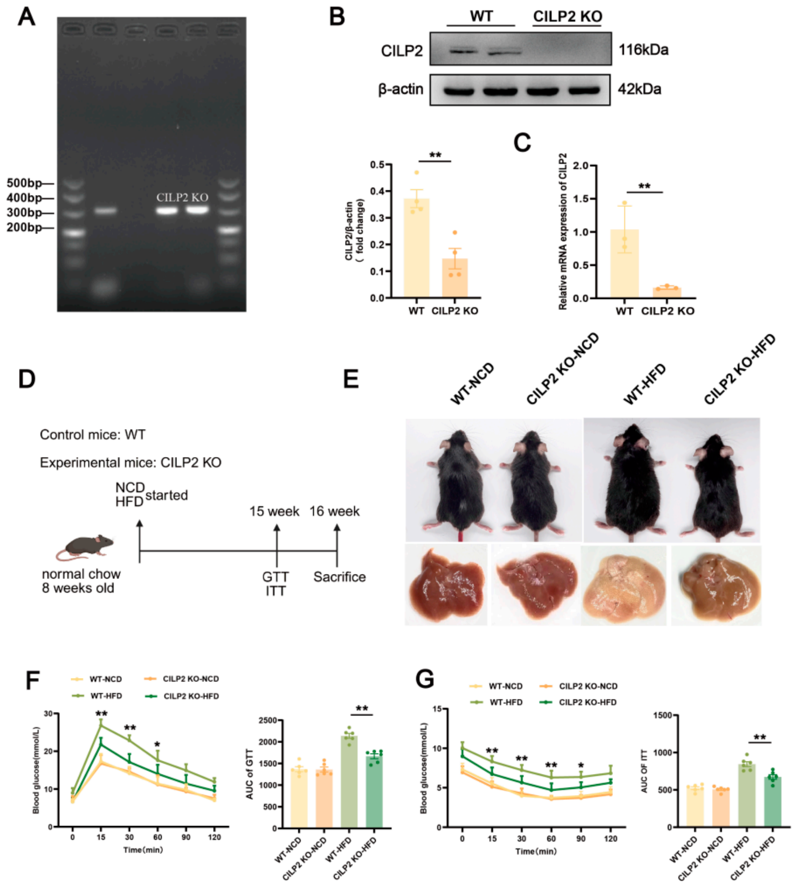

Figure S1. CILP2 KO mice construction

(A)Genotype identification. (B) Immunoblots (upper) and quantification (lower) of hepatic CILP2 in the WT or CILP2 KO mice (n=4). *P<0.05; two-tailed unpaired Student's t test. (C) qPCR analysis of Cilp2 mRNA expression in the WT or CILP2 KO mice (n=3). **P<0.01; two-tailed unpaired Student's t test. (D)The schematic flow. (E) Representative images of mice and liver in the WT or CILP2 KO mice fed with NCD or HFD for 16 weeks. (F) Right: AUC of GTT. Left: GTT in the WT or CILP2 KO mice (n=6). *P<0.05,**P<0.01; two-tailed unpaired Student's t test. (G) Right: AUC of ITT. Left: ITT in the WT or CILP2 KO mice (n=6). *P<0.05,**P<0.01; two-tailed unpaired Student's t test.

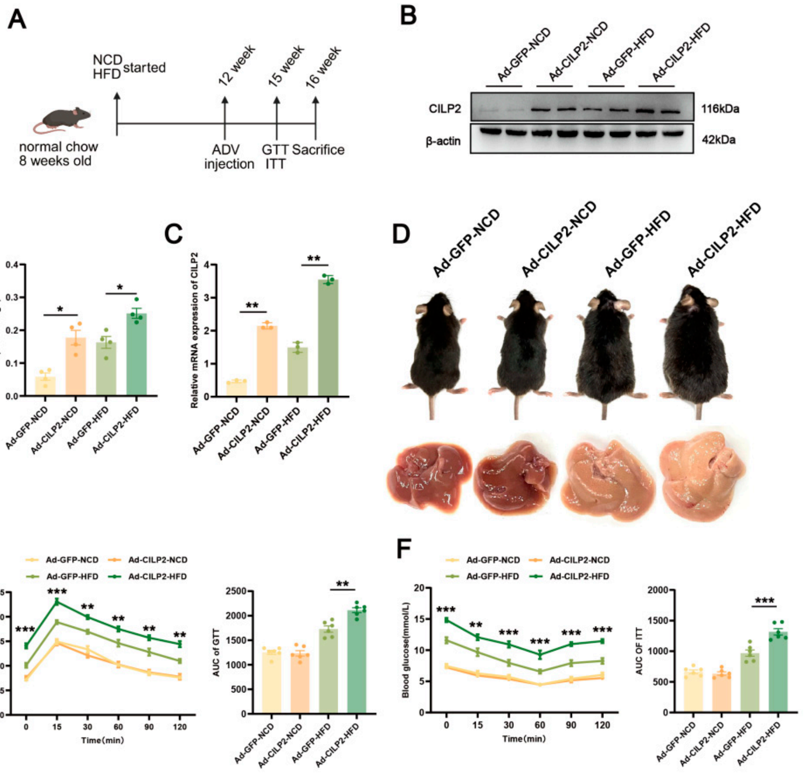

Figure S2. CILP2 overexpression in mice

(A) The schematic flow. (B) Immunoblots (upper) and quantification (lower) of hepatic CILP2 protein level in the Ad-GFP or Ad-CILP2 mice (n=4). *P<0.05; one-way ANOVA with multiple comparisons. (C) qPCR analysis of CILP2 mRNA expression in the Ad-GFP or Ad-CILP2 mice (n=3). **P<0.01; one-way ANOVA with multiple comparisons. (D) Representative images of mice and liver in the Ad-GFP or Ad-CILP2 mice fed with NCD or HFD for 16 weeks. (E) Right: AUC of GTT. Left: GTT in the Ad-GFP or Ad-CILP2 mice (n=6). **P<0.01, ***P<0.001; two-tailed unpaired Student's t test. (F) Right: AUC of ITT. Left, ITT in the Ad-GFP or Ad-CILP2 mice (n=6).**P<0.01, ***P<0.001; two-tailed unpaired Student's t test.

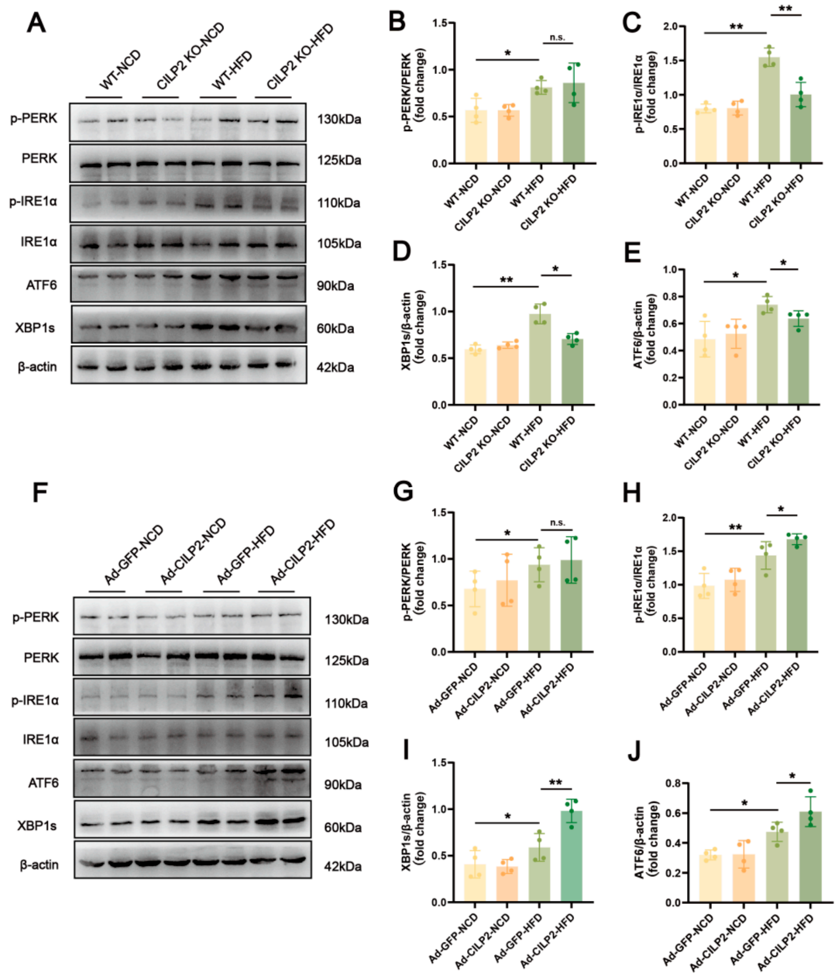

Figure S3. CILP2 promoted the activation of the IRE1α/XBP1 pathway in vivo.

(A-E) Immunoblots (A) and quantification of hepatic p-PERK (B), p-IRE1α (C), XBP1s (D) and ATF6 (E) protein levels in the WT or CILP2 KO mice fed with NCD or HFD for 16 weeks (n=4). *P<0.05, **P<0.01; one-way ANOVA with multiple comparisons. (F-J) Immunoblots (F) and quantification of hepatic p-PERK (G), p-IRE1α (H), XBP1s (I) and ATF6 (J) protein levels in the Ad-GFP or Ad-CILP2 mice fed with NCD or HFD for 16 weeks (n=4). *P<0.05, **P<0.01; one-way ANOVA with multiple comparisons.
